# Isochalcogenourea-catalyzed asymmetric (4 + 2)-heterocycloadditions of allenoates

**DOI:** 10.1093/chemle/upae168

**Published:** 2024-08-23

**Authors:** Mario Waser

**Affiliations:** Institute of Organic Chemistry, Johannes Kepler University Linz, Altenbergerstrasse 69, 4040 Linz, Austria

**Keywords:** Allenoates, cycloadditions, Lewis base catalysis, organocatalysis

## Abstract

Allenoates are versatile reagents that can be used for numerous (formal) cycloaddition reactions under (chiral) Lewis base catalysis. Most commonly, the catalysts of choice are phosphines, amines, and *N*-heterocyclic carbenes. We have recently established the use of readily available chiral isochalcogenoureas as catalysts for asymmetric (4 + 2)-heterocycloadditions of allenoates with various vinylogous acceptors. This represents a complementary approach for allenoate activation and gives access to various highly functionalized chiral dihydropyrans with good to excellent enantioselectivities and diastereoselectivities.

## Graphical abstract

**Graphical Abstract:**
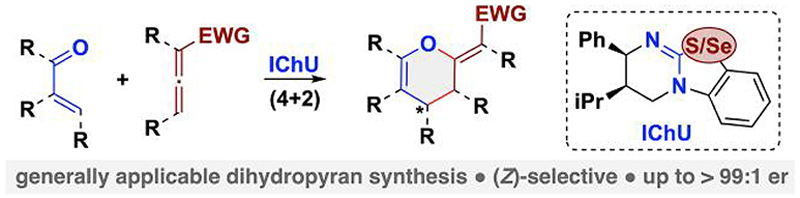
Allenoates are versatile reagents that can be used for numerous (formal) cycloaddition reactions under (chiral) Lewis base catalysis. Most commonly, the catalysts of choice are phosphines, amines, and *N*-heterocyclic carbenes. We have recently established the use of readily available chiral isochalcogenoureas as catalysts for asymmetric (4 + 2)-heterocycloadditions of allenoates with various Michael acceptors. This represents a complementary approach for allenoate activation and gives access to products that are not easily accessible when using the established catalyst systems.

## 1 Allenoates—established reactivities

**Scheme 1:**
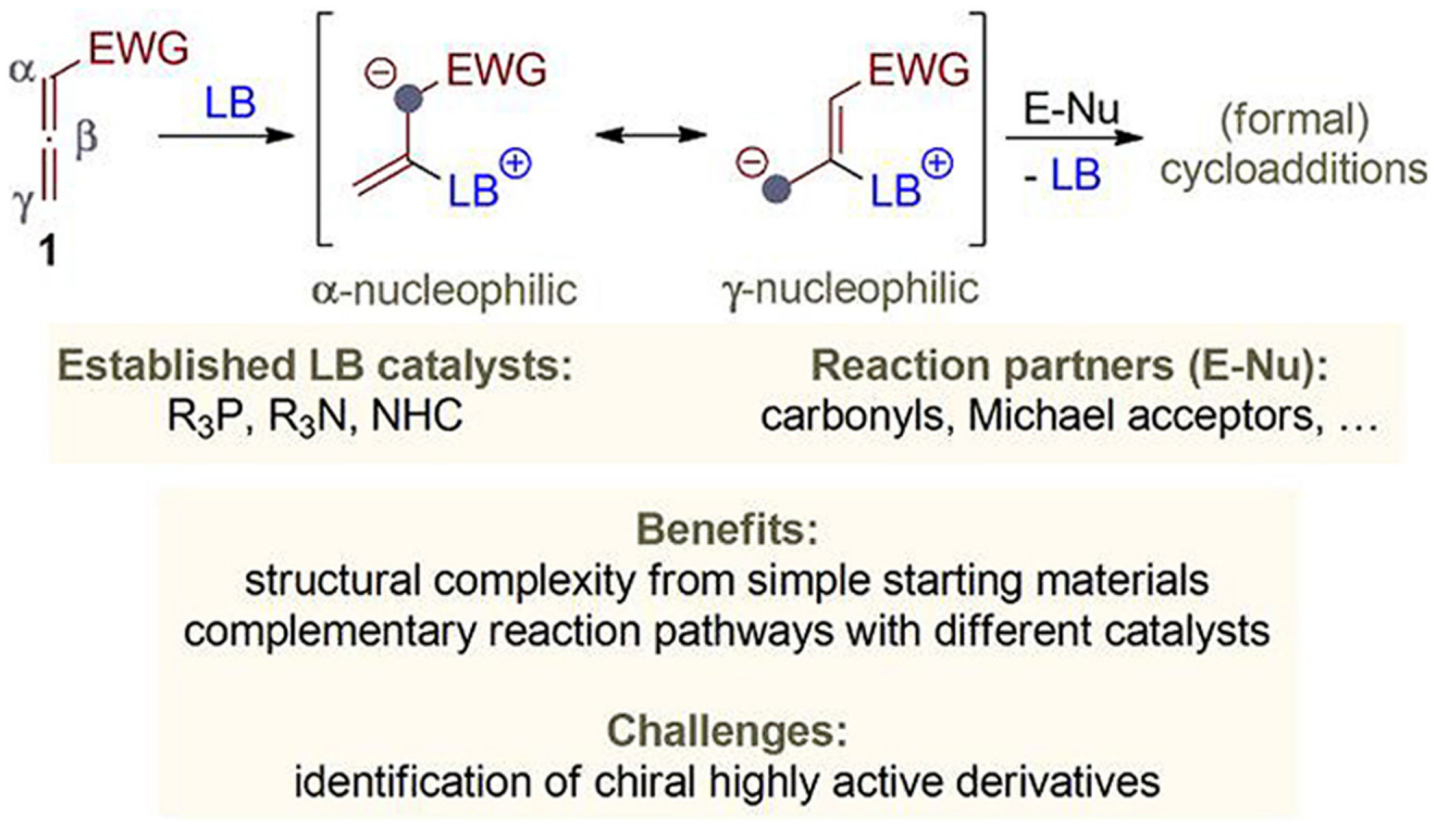
General overview on Lewis base-catalyzed (formal) cycloadditions of allenoates.

Allenoates **1**, the esters of buta-2,3-dienoic acid, and analogous ketones or nitriles are easily accessible building blocks with extraordinary reactivities.^1^ These cumulated dienes can undergo a multitude of different transformations, in either a non-catalyzed or a catalyzed manner. With respect to the latter, especially the use of (chiral) nucleophilic Lewis base (LB) organo-catalysts^2^ has allowed the introduction of a broad variety of structural complexity-generating approaches.^1,3–7^ Activation hereby happens in a covalent manner by addition of the LB to the sp-hybridized β-carbon of the allenoate, thus resulting in the formation of highly reactive resonance-stabilized betaine species first (Scheme 1). Depending on the catalyst used as well as the decoration of the employed allenoate, either the α-or the γ-carbon may be the most nucleophilic position. Furthermore, in the case of α- or γ-substituted allenoates, the side chains may also become nucleophilic after a proton transfer. These betaines can then add to a variety of different acceptor molecules in a stereoselective fashion when chiral catalyst derivatives are used.^1,3,5–7^ Most commonly, potentially dipolar cycloaddition partners (E-Nu) such as carbonyl derivatives or Michael-type acceptors are used. In such cases, the initial addition of the LB-activated allenoate to these reagent leads to the formation of nucleophilic intermediates such as alcoholates or enolates. As a consequence, these primary addition products can then undergo ring-closure by “back-biting” to the allenoate skeleton with subsequent release of the catalyst, thus allowing catalytic turnover. Accordingly, this concept holds enormous potential for the access of complex and structurally diverse chiral carbo- and heterocycles from simple prochiral starting materials using chiral organocatalysts. With respect to the catalyst motifs of choice, the field is mainly dominated by (chiral) tertiary phosphines^3,4^ and, to a lesser extent, tertiary amines^26^ and *N*-heterocyclic carbenes.^3,4^ Very importantly, the nature of the used catalyst has a pronounced effect on the reactivity of the betaines in such (formal) cycloaddition reactions. This often allows orthogonal reaction pathways when changing the catalyst principle.^7^ Unfortunately, however, the identification of chiral catalyst derivatives that allow both high catalyst turnover and excellent enantioselectivities can sometimes be challenging.^8^ One reason therefore can be a potentially lower reactivity (nucleophilicity, Lewis basicity) of the usually sterically more demanding chiral derivatives as compared with simple achiral ones (which sometimes have to be used in almost stoichiometric quantities already). In addition, especially P(III)-compounds may show a pronounced sensitivity toward oxidation. Therefore, the exploration of alternative (bench-stable) catalyst motifs to render allenoate reactions enantioselective would be a worthwhile goal, as this may allow complementary novel reaction pathways with high catalytic efficiencies.

## 2 Isochalcogenoureas—established activation modes

**Scheme 2:**
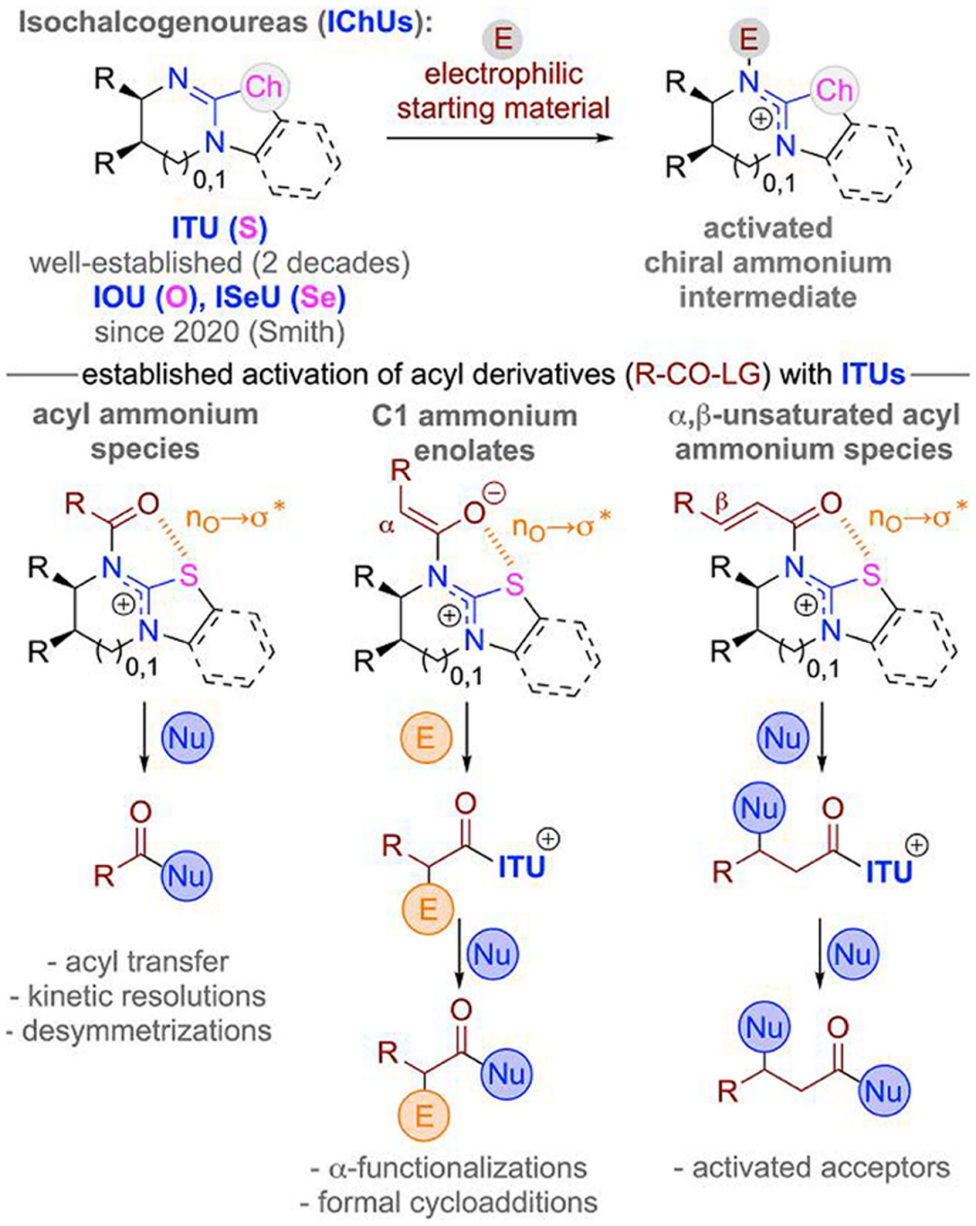
Established activation modes of isochalcogenoureas.

Isochalcogenoureas (IChUs, Scheme 2) are a class of versatile amidine derivatives that contain an organochalcogen group attached to the sp^2^-carbon of the amidine functionality.^9^ This chalcogen group significantly influences the basicity and nucleophilicity of the amidine motif and the corresponding sulfur derivatives—the so-called isothioureas (ITUs)—have found widespread use as powerful easily accessible LB organocatalysts over the course of the last 2 decades (Scheme 2).^9,10^ Pioneering work by Birman’s group revealed the high potential of these bench-stable heterocycles as chiral acyl transfer-reagents.^11,12^ Over the years, a multitude of efficient (asymmetric) acyl transfer-based reactions such as (dynamic) kinetic resolutions of alcohols, azlactones, (thio)lactames, oxazolidinones, azirines, and carboxylic acid derivatives using ITU catalysts have been developed.^9^ Apart from these acylation-type reactions (Scheme 2, left column), ITUs have been very successfully used for the generation and utilization of chiral C1 ammonium enolates^13–15^ and chiral α,β-unsaturated acyl ammonium species.^16^ All these applications rely on the initial addition of the ITU to an activated carboxylic acid derivative (e.g., acyl halides, anhydrides, electron-deficient esters). The hereby formed ammonium species benefit from a very well-defined synperiplanar conformation because of a stabilizing 1,5-O--S (n_O_->σ*) interaction,^9,12b,13,17–19^ thus allowing high levels of enantioselectivities in a highly predictable manner for the follow-up transformations. More specifically, the C1 ammonium enolates can undergo various α-functionalizations followed by the release of the catalyst via the addition of a nucleophilic species (either in a bimolecular manner or via a formal cycloaddition).^9,13–15,20^ The application of this powerful LB catalysis concept has led to the development of a broad variety of highly efficient asymmetric strategies for carbonyl α-functionalizations and cycloadditions over the course of the last decade.^13,14,20^ Furthermore, it was also recently demonstrated that this enolate-activation concept can be successfully combined with complementary catalysis modes (e.g., transition metal catalysis).^15^ The α,β-unsaturated acyl ammonium species, on the other hand, serve well as Michael acceptors and the catalyst is then again released by the addition of a second nucleophilic species.^16^ Although this strategy has not been as exhaustively exploited as the afore-mentioned acylations and C1 ammonium enolate activations so far, a variety of very appealing applications have been reported already.^16^ Furthermore, it was recently also shown that these α,β-unsaturated acyl ammonium species can be successfully engaged as acceptors for asymmetric 1,4-additions of radical nucleophiles.^14c,21^

In 2020, Smith’s group succeeded in introducing first derivatives of analogous O- and Se-derivatives (IOUs and ISeUs).^22^ In this seminal contribution, a pronounced effect of the chalcogen on reactivity, catalytic activity, and the 1,5-O--Ch interaction was observed. ISeUs showed a stronger n_O_->σ* interaction and higher catalytic reactivity in all the investigated test reactions (covering the 3 different activation modes) compared with ITUs, which reacted significantly more slowly, albeit with similar levels of enantioselectivities. IOUs were the least reactive and also did not show any n_O_->σ* stabilization, thus resulting in low levels of enantioselectivities. Overall, these remarkable observations have paved the way for a conceptually unique and powerful tool for tuning the reactivity of these LB amidine-type catalysts, and recent studies by Smith’s group and ourselves have already shown that ISeUs can be very efficiently used to increase reaction rates (and in some cases even enantioselectivities) in target transformations where the established ITUs may react more slowly.^22,23^

With respect to the practical use of these catalysts, it should also be emphasized that several studies have shown that ITUs can be easily immobilized on different solid supports^24^ and it therefore seems to be only a question of time until solid-supported ISeUs will be available as well.

## 3 Isochalcogenoureas—allenoate activation

Considering the high catalytic potential of IChUs as chiral LB organocatalysts on the one hand and the versatility of allenoates to undergo a multitude of complex (formal) cycloaddition reactions in the presence of chiral LBs on the other, we recently wondered whether chiral IChUs may be successfully employed to control allenoate reactions.^25,26^ Surprisingly, and to the best of our knowledge, this reagent/catalyst combination had not been systematically explored until then. Thus, we started to investigate the possibility of carrying out reactions between allenoates **1** and different Michael-type acceptors in the presence of established IChUs. Hereby, we were especially interested in addressing the following questions:

- Do IChUs in general have the potential to activate allenoates?
- Are complementary reaction pathways accessible compared with established catalyst motifs?
- Can these transformations be carried out enantioselectively?

**Scheme 3:**
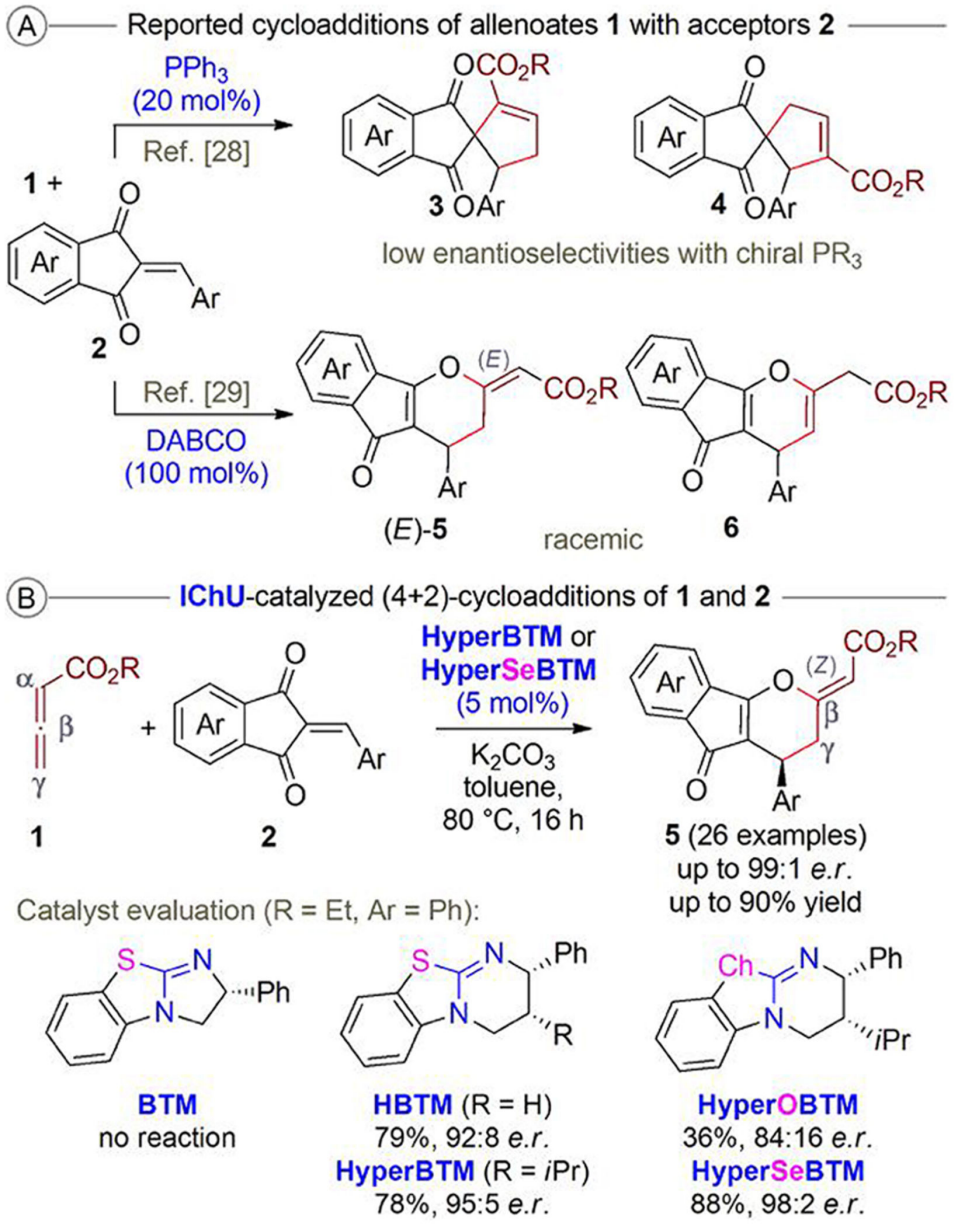
a) Cycloadditions of allenoates **1** with acceptors **2** in the presence of phosphines and amines and b) our recently developed (4 + 2)-cycloaddition using IChUs.

Our first test reaction was the cycloaddition of allenoates **1** with the benzylidene-indandiones **2** (Scheme 3).^25^ Acceptors **2** were chosen because it was recently demonstrated by other groups that they can undergo cycloadditions with allenoates **1** in the presence of phosphines and amines (Scheme 3a).^27–29^ Remarkably, while the use of PPh_3_ leads to the formation of the cyclopentene-based products **3** and **4** in a (3 + 2)-manner,^28^ the use of DABCO (1,4-diazabicyclo[2.2.2]octane) favors the (4 + 2)-cycloaddition products (*E*)-**5** and **6** (preference for the endo- or exo-cyclic double bond depends on the reaction conditions).^29^ While a first proof-of-concept for an enantioselective variant using chiral phosphines was obtained (albeit with rather low selectivities only),^28b^ the DABCO protocol required a stoi-chiometric amount of amine and could be carried out in a racemic manner only.^29^ As a consequence, we thought that this starting material combination would be an interesting test reaction in order to establish our targeted IChU-catalyzed allenoate cycloadditions.

Very encouragingly, we quickly realized that well-established achiral and chiral ITUs can indeed be used to activate allenoates for cycloaddition reactions. Interestingly, however, higher reaction temperatures of around 80 °C were necessary, as we observed only a rather slow and non-productive consumption of the starting materials at room temperature. Elevated temperatures, on the other hand, allowed the efficient formation of the (*Z*)-configurated dihydropyrans **4** in high yields and with only traces of regioisomers that originated from the α-attack of the allenoate (Scheme 3b). In principle, amine and phosphine-catalyzed (4 + 2)-cycloaddition reactions of allenoates to access dihydropyran derivatives are well known^30^ but, in most cases, these strategies deliver products with an (*E*)-configurated exocyclic double bond. In addition, as outlined for the DABCO-activated reaction of **1** and **2** (Scheme 3a),^29^ these approaches may sometimes reach their limits with respect to catalyst loading and when enantioselective variants are targeted. Accordingly, we were very delighted to see that our initial hypothesis that IChUs are capable of activating allenoates in a complementary way as compared with tert. phosphines and tert. amines was indeed valid, and that this strategy allows the catalytic synthesis of targets that are not easily accessible when using the established catalysis systems. In addition, we were excited to see that the use of chiral IChUs actually allowed rather high levels of enantioselectivities (Scheme 3b). Remarkably, both the nature of the chalcogen and also the parent scaffold of the catalyst had a significant influence on the reactivity, which can most likely be attributed to their different nucleophilicities and Lewis basicities.^10^ While the 5-ring-based BTM did not allow any product formation, the 6-ring-based HBTM and HyperBTM gave the products in good yields and with high selectivities. The isourea derivative HyperOBTM was also clearly less reactive as compared with HyperBTM and the selenium-analog HyperSeBTM, which was found to be the most reactive one. The same reactivity trend was also recently observed by Smith’s group when they investigated these catalysts for acylations, C1 ammonium enolate reactions, and the activation of α,β-unsaturated acceptors.^22^ With respect to the observed enantioselectivities, the “Hyper”-scaffold was found to be the best, with the Se-variant (HyperSeBTM) being slightly more selective than the classical S-based HyperBTM. Overall, this approach allowed us to access a variety of highly functionalized dihydropyrans **5** with good to excellent enantioselectivities, exclusive (*Z*)-configuration of the exocyclic double bond, and in high yields. In addition, we also tested a few γ-alkyl-substituted allenoates that were equally well accepted and gave the tricyclic products **5** (containing an additional alkyl group in the γ-position), too.^25^ The application scope was investigated by using both HyperBTM and HyperSeBTM, and, in almost all cases, the Se-derivative was slightly more reactive and more selective, and it was also possible to reduce the catalyst loading to 1 mol% without significantly affecting the outcome.^25^

**Scheme 4:**
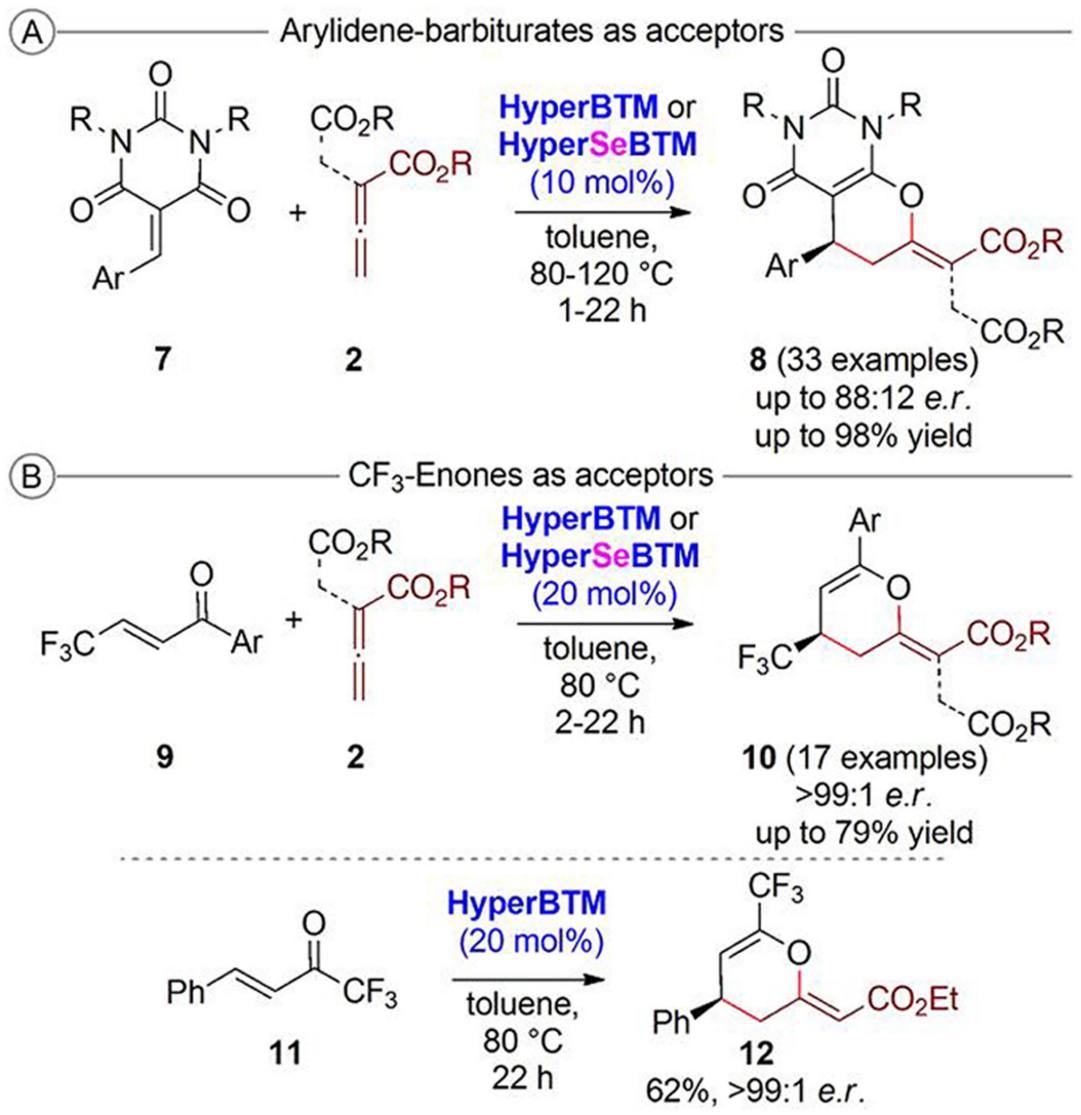
IChU-catalyzed (4 + 2)-cycloadditions using different Michael acceptors and allenoates.

With this first proof-of-concept for the high and, until then, uncovered potential of IChUs to activate the allenoates at hand, we next investigated alternative acceptors to demonstrate the generality of this (4 + 2)-cycloaddition strategy (Scheme 4).^26^

By testing the arylidene-barbiturates **7** as acceptors, we realized that they reacted in the same (4 + 2)-manner as compounds **2**, delivering the dihydropyran products **8** with a (*Z*)-configurated exocyclic bond as well (Scheme 4a). Unfortunately, enantioselectivities were slightly lower as compared with those of the syntheses of compounds **5** and higher catalyst loadings were required, too (compare with Scheme 3b). Interestingly, when α-branched allenoates **2** were used, the side chain did not participate at all in the cycloaddition process and instead turned out to be an “innocent” substituent in this case. This result is in sharp contrast to our recent observations when using such allenoates under chiral phosphine catalysis in combination with quinone methides, where this side chain turned out to be the reactive center.^8^ Using the CF_3_-enones **9** allowed the analogous (4 + 2)-cycloaddition as well, giving the products **10** with excellent enantioselectivities and in good yields when using 20 mol% of the catalysts (Scheme 4b). In both cases, for syntheses of compounds **8** and **10**, the HyperBTM catalyst scaffold was the best-suited one and again the ISeU derivative was slightly more active and selective as compared with the ITU. We also carried out quick screening using the CF_3_-enone **11**, which reacted in the analogous highly selective (4 + 2)-manner, but unfortunately in lower yield. In addition, further substrate variations were not well tolerated, as these compounds underwent partial decomposition under the reaction conditions. It is noteworthy that this decomposition was more pronounced when using the Se-based catalyst, thus again underscoring the higher reactivity (nucleophilicity) of the Se-congener. Nevertheless, all these investigations clearly demonstrate that our IChU-catalyzed (4 + 2)-cycloaddition of allenoates is a rather general process that tolerates different Michael acceptors. Furthermore, in all the cases in which we have been able to assign the absolute configuration of the products, we observed the same sense of induction.

**Scheme 5:**
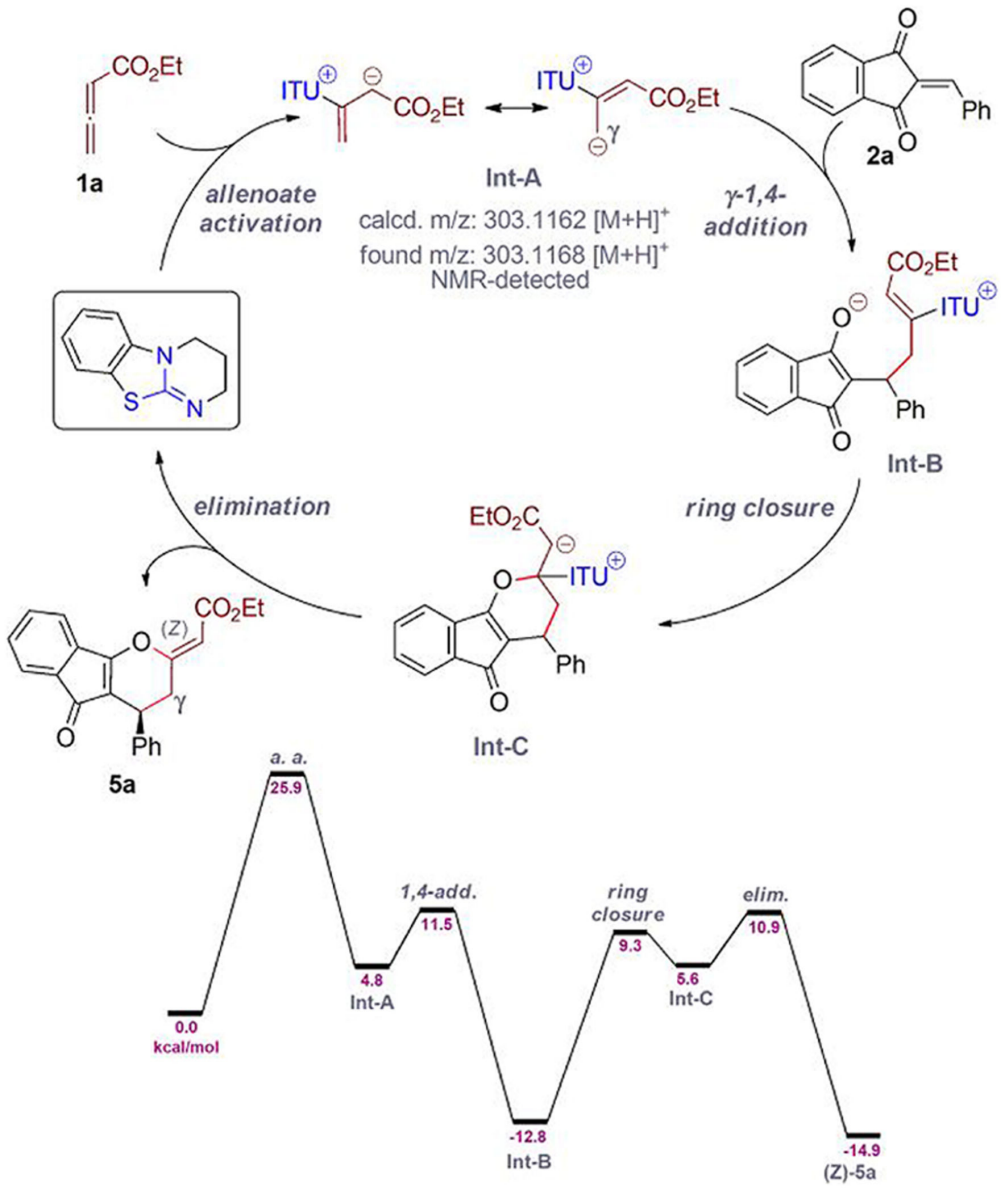
Mechanistic details of our IChU-catalyzed (4 + 2)-cycloaddition of allenoates.

With all these results demonstrating the broader applicability of this concept at hand, questions concerning the reaction pathway arose. Furthermore, we were also wondering whether density functional theory calculations could provide an explanation for the orthogonal cycloaddition paths observed with different catalyst classes. Thus, we teamed up with Raphaël Robiette (Belgium), who carried out detailed computational studies of the reaction of allenoates **1** with the benzylidene-indandiones **2**.^25^ The calculations were first carried out by using an achiral ITU derivative to obtain a detailed understanding of the individual steps and then with chiral isothiourea derivatives as well as PPh_3_ and DABCO. As outlined in Scheme 5, the sequence commenced with β-addition of the ITU to the allenoate, giving the resonance-stabilized betaine **Int-A**. Formation of this intermediate could be detected by using HRMS and ^1^H NMR. The γ-anionic species was found to be more nucleophilic and underwent 1,4-addition to the acceptor delivering **Int-B**. This is the step in which the chiral catalyst derivatives control the formation of the newly formed stereogenic center and our calculations are in accordance with the experimentally observed sense of induction and level of enantioselectivity. The next step was the ring-closure to **Int-C**. This step is the same when using ITU and DABCO, while PPh_3_ favors 5-ring formation instead (compare with Scheme A—compounds **3** and **4**) because of the generation of a rather stable phosphonium ylide intermediate in this case.^25^ Finally, catalyst-elimination sets the exocyclic double bond of products **5** with kinetic preference for the (Z)-diastereomer when using ITUs as catalysts.

With respect to the energy barriers of the individual steps, we found that the addition of the catalyst to the allenoate was the rate-limiting step in this case. In addition, the ring-closure/elimination sequence from **Int-B** to the final product was found to be high in energy. We rationalize that these 2 energetically demanding steps are the reasons for the required elevated reaction temperatures and why the various catalyst derivatives perform differently. As the first step strongly depends on the activity of the catalyst, more nucleophilic/basic catalyst variants should be better suited for allenoate activation. Previous studies by Smith and Mayr^10^ have shown that the catalyst scaffold, namely the ring size of the amidine part, influences the nucleophilicity (*N*-parameter) and Lewis basicity. Not surprisingly, the 6-ring amidine-containing HyperBTM and HBTM that were suited in our case (Scheme 3b) show higher *N*-parameters as derivatives with a 5-ring amidine motif (it should be stated that BTM, which was not active in our case, was not part of this study^10^ but 5-ring-based achiral derivatives were less nucleophilic as compared with the homologous 6-ring derivatives). Furthermore, the higher reactivity of HyperSeBTM compared with HyperBTM can also most likely be explained by its higher nucleophilicity/Lewis basicity (compare with Ref. ^21^). Furthermore, the leaving group quality of the catalyst can also not be ignored considering the high barrier from **Int-B** to the final product and it may be that, depending on the nature of the catalyst, this step becomes rate-determining.

Overall, we think that we could demonstrate that IChUs are indeed very well-suited catalysts for the activation of allenoates. These easily accessible and bench-stable Lewis bases allow orthogonal reaction pathways as compared with other LB classes and have given good to excellent selectivities in applications so far. We are therefore confident that they will serve well for future applications in this direction and further studies are ongoing in our laboratory.
